# Immunomodulatory functional foods and their molecular mechanisms

**DOI:** 10.1038/s12276-022-00724-0

**Published:** 2022-01-25

**Authors:** Jae Hwan Kim, Da Hyun Kim, Seongin Jo, Min Je Cho, Ye Ryeong Cho, Yong Joon Lee, Sanguine Byun

**Affiliations:** Department of Biotechnology, Yonsei University, Seoul, 03722 Korea

**Keywords:** Health care, Pathogenesis

## Abstract

The immune system comprises a complex group of processes that provide defense against diverse pathogens. These defenses can be divided into innate and adaptive immunity, in which specific immune components converge to limit infections. In addition to genetic factors, aging, lifestyle, and environmental factors can influence immune function, potentially affecting the susceptibility of the host to disease-causing agents. Chemical compounds in certain foods have been shown to regulate signal transduction and cell phenotypes, ultimately impacting pathophysiology. Research has shown that the consumption of specific functional foods can stimulate the activity of immune cells, providing protection against cancer, viruses, and bacteria. Here, we review a number of functional foods reported to strengthen immunity, including ginseng, mushrooms, chlorella, and probiotics (*Lactobacillus plantarum*). We also discuss the molecular mechanisms involved in regulating the activity of various types of immune cells. Identifying immune-enhancing functional foods and understanding their mechanisms of action will support new approaches to maintain proper health and combat immunological diseases.

## Functional foods: Beneficial effects on the immune system

Evidence is building to support the idea that specific ‘functional foods’ can stimulate the activity of cells and signaling systems of the immune system to provide protection against cancer, viruses and bacteria. Sanguine Byun and colleagues at Yonsei University in Seoul, South Korea, review research into a range of functional foods, foods thought to have health benefits beyond their nutritional value. These include ginseng, mushrooms, the green algae called *Chlorella* and the probiotic bacteria *Lactobacillus plantarum*. They also consider individual components of foods such as poly-gamma-glutamate, a natural polymer made by bacteria. A wide body of research is revealing diverse molecular mechanisms through which biochemicals in functional foods can modulate different aspects of the immune system. These include effects on both non-specific innate immunity and adaptive immunity, which targets specific invading pathogens and diseased cells.

## Introduction

The immune system is a network composed of various structures and biological processes that defend the host against pathogens. Impairment of the immune system affects the susceptibility of the host to foreign pathogens and may lead to diseases such as cancer and viral infections^1^. A study reported that the response to vaccines is significantly reduced in elderly individuals, as immune function declines with age^2^. On the other hand, immune enhancement positively correlates with lower cancer incidence. According to a cohort study, individuals with higher lymphocyte cytotoxic activity had a reduced risk of cancer^3^. With the increase in life expectancy, concerns about the age-mediated weakening of immune functions are considered an important social health issue^4^. In addition, lifestyles, dietary patterns, and environmental hazards can also affect immunity, further emphasizing the importance of maintaining a healthy immune system^5–7^. Certain foods have been shown to have immunostimulatory effects, providing protection against microbial pathogens and cancer progression^8^. In this review, we discuss functional foods reported to improve immunity as well as their molecular mechanisms of action.

## The immune system

### Innate immunity

The immune system can be grouped into two categories: innate immunity and adaptive immunity. The innate defense system is an immediate nonspecific response mediated by various types of immune cells, including macrophages, natural killer (NK) cells, and dendritic cells (DCs)^9^. Macrophages are essential cells of the innate immune system that can remove pathogens through phagocytosis and subsequently recruit other immune cells to fight against invaders^10^. Additionally, activated macrophages secrete cytokines such as tumor necrosis factor (TNF)-α, which acts as a meditator for activating/recruiting NK cells, neutrophils, and eosinophils^11,12^. In addition to cytokine secretion, nitric oxide (NO) production by inducible NO synthase (iNOS) is a method that macrophages use to destroy foreign microbial agents^13^. Toll-like receptors (TLRs) are pattern recognition receptors that play an important role in the regulation of the immune system by macrophages. Activation of TLR2, which in turn induces mitogen-activated protein kinase (MAPK) signaling pathways, and nuclear factor κ-light-chain-enhancer of activated B cells (NF-κB) has been known to be a major mechanism controlling the immune response in macrophages^14–16^. NK cells play a pivotal role in surveillance and defense against virus infection and malignant cells. NK cells secrete interferon (IFN)-γ as a signal to activate macrophages for phagocytosis, further augmenting the immune response^17^. DCs originate from hematopoietic bone marrow progenitor cells. DCs are professional antigen-presenting cells that link the innate and adaptive immune systems by processing antigens and presenting them to T lymphocytes^18^. Overall, these dedicated immune cells are involved in the first line of defense against external microbes as a part of the innate immune system, with a role in identifying nonself elements and generating cytotoxic effects.

### Adaptive immunity

Adaptive immunity is an antigen-specific defense system characterized by the activity of B and T cells. The adaptive immune response takes much longer than the innate immune response but is more specific to the pathogen and uses immunological memory to enhance the response when re-exposed in the future. B cells produce unique antibodies in response to antigens of invading pathogens. The binding of antibodies to specific antigens can neutralize the pathogen directly and/or activate macrophages to phagocytose foreign entities. These antibodies also promote the formation of the complement system on the microbe’s membrane to initiate destruction^19^.

Cytotoxic T cells directly kill host cells that harbor foreign molecules, while helper T (T_H_) cells enhance the immune response by controlling the activity of other immune cells, such as B cells and cytotoxic T cells. Proper differentiation of naive T cells into specific types of T cells after exposure to viral or bacterial antigens is crucial for fine-tuning the immune response against antigenic challenge^20^. Cytotoxic T cells induce cell death of victim cells by cell-mediated destruction, which requires direct physical contact. Cytotoxic T cells release granzymes and perforins, which disrupt membrane integrity and trigger apoptosis of the target cell. Additionally, the Fas ligand (FasL) expressed on the surface of cytotoxic T cells binds to the Fas receptor of the target cell, causing apoptosis of the target cell via the caspase cascade^21^. T_H_ cells communicate with both B cells and T cells. T_H_ cells are divided into several subsets, of which T_H_ 1 cells play an important role in regulating cell-mediated responses related to cytotoxic T cells and macrophages^22^. T_H_ 1 cells produce TNF-α, IL-12, IL-2, or IFN-γ to induce cellular immunity and are related to defense against intracellular microbes. T_H_ 2 cells participate in protection against parasites and produce IL-4, IL-5, and IL-10 to orchestrate immune responses, such as the control of B cells^23^. T_H_ 17 cells defend against pathogens by secreting IL-6, IL-17, or IL-22 and are involved in host defense against bacteria and fungi^24,25^.

## *Panax ginseng* C.A. Meyer (Ginseng)

Ginseng is one of the most well-known medicinal foods and has been studied for its immunostimulatory effects^26^. Ginseng has been widely consumed in two major forms: white ginseng and red ginseng. White ginseng is produced by dehydration of ginseng, while red ginseng is produced by steaming and drying raw ginseng multiple times^27,28^. Ginseng roots as a whole, as well as their constituents, have been studied as immunostimulants (Table 1).

**Table 1:** The immunomodulatory functions and mechanism of ginseng.

Source	Cell type	Function	Model	Reference
White ginseng	PBMCs	IL-12, IFN-γ↑	Animal	^38^
	Splenocytes	IgA, IgG, IgM, IL-2, IL-4, IL-10, IFN-γ, TNF- α↑	Animal	^43,48,49^
		IgG1, IgG2a, IL-2, IL-4, IL-5, IL-6, IL-10, TNF- α, IFN-γ,	Animal	^58,60^
	Macrophages	Phagocytosis, NO, iNOS↑ IL-1α, IL-1β, IL-6, IL-23↑ COX-2, TNF- α, JNK↑	In vitro	^31–33,35,36^
	NK cells	Cytotoxicity↑	Animal,human	^43–45^
	T cells	Cell proliferation↑	Human	^45^
Ginsan	Splenocytes	IgG1, IgG2, IgA, COX-1 ↑ T cell proliferation↑ IL-1, IL-6, IL-12, IFN-γ↑	Animal	^51,53^
	Macrophages	NO, iNOS, IFN-γ, TLR-2↑ IL-1β, IL-6, IL-12, IL-18, TNF- α↑	Animal	^40,41^
	CD4+ T cells	IL-4, IFN-**γ** ↑	Animal	^39^
	DCs	IL-12, IFN-γ, TNF- α↑ MHC class II, CD86↑	Animal	^39^
Red ginseng	PBMCs	CD25, CD69 expression↑	Ex vivo	^63^
	Macrophages	NO, iNOS, IL-1, IL-6↑	In vitro	^64,135^
	NK cells	Cytotoxicity, IFN-γ, NKp46↑	Animal	^63^
		CD25, CD69 expression↑ Cell proliferation↑	Ex vivo	^63^
	Blood	IgA, all types of IgG, IFN-γ↑	Animal	^63,65,68^
		T cells, B cells, WBCs↑	Human	^74^
		ARI frequency rate↓	Human	^75^
Ginsenoside Rg1	Splenocytes	Cell proliferation↑ IL-4, IL-10, IL-12, IFN-γ↑	Animal	^50,71^
	Macrophages	NF-κB, IL-1, IL-2↑	Animal	^46,71^
	NK cells	Cytotoxicity↑	Animal	^46^
	CD4+ T cells	IL-2, IL-4, IL-10, IL-12, IFN-γ↑	Animal	^54,56^
	Blood	IgG1, IgG2a↑	Animal	^50^
Ginsenoside Rd	Splenocytes	Cell proliferation↑ IL-2, IFN-γ, IL-4, IL-10↑	Animal	^69^
	NK cells	Cytotoxicity↑	In vitro	^55^
	T cells	T_H_ 2 cell proliferation↑	In vitro	^55^
	Blood	IgG, IgG1, IgG2b↑	Animal	^69^
Ginsenoside Re	Splenocytes	Cell proliferation↑ IL-4, IL-5, IL-10, IL-12, IFN-γ↑	Animal	^52,70,71^
	Macrophages	NF-κB↑	Animal	^71^
	Blood	IgG, IgG1, IgG2a, IgG2b↑	Animal	^70,71^
Ginsenoside Rb1	Splenocytes	IL-2, IL-4, IL-10, IFN-γ, TNF- α↑ IgG1, IgG2a, IgG2b↑	Animal	^57^
Ginsenoside Rc	NK cells	Cytotoxicity↑	In vitro	^55^
	T cells	T_H_ 2 cell proliferation↑	In vitro	^55^

### White ginseng

White ginseng is reported to contain various immunomodulating components, including ginsenosides and polysaccharides^29,30^. The innate immunostimulatory effects of white ginseng extracts and their constituents have been extensively studied, focusing on macrophages, DCs, and NK cells. Treatment with white ginseng extract increased the phagocytic activity of RAW 264.7 murine macrophages and dose-dependently upregulated iNOS expression and NO production^31–33^. White ginseng extracts also enhanced the expression of proinflammatory cytokines, including IL-6, IL-1β, and TNF-α, in RAW264.7 cells^33,34^. In addition to cytokine secretion, Lim et al.^31^ demonstrated that white ginseng extract stimulated the recruitment of immune cells to the site of infection and increased the expression of the proinflammatory cytokines TNF-α, IL-1α, and IL-23 in RAW 264.7 macrophage cells through the activation of the MAPK kinase (MKK)4-c-Jun N-terminal kinase (JNK)-c-Jun signaling pathway. The phosphorylated levels of JNK1, JNK2, and ERK2 were found to increase upon administration of white ginseng extracts^31^. Pretreatment with JNK inhibitors in white ginseng extract-activated RAW264.7 cells significantly reduced the production of immunomodulators such as NO, IL-6, and TNF-α^31^. This result confirms that the immunomodulatory effects of white ginseng extracts are dependent on the activation of the MAPK/JNK pathway. Previous studies reported that MAPK, NF-κB, and PI3K/AKT signaling are dependent on TLR2/4-mediated immune responses^35,36^. Um et al.^33^ revealed that treatment with white ginseng extract in RAW 264.7 cells enhanced macrophage phagocytosis through TLR2/4-dependent activation of the MAPK, NF-κB, and PI3K/AKT signaling pathways. Um et al.^33^ further demonstrated that white ginseng extract-induced activation of NF-κB and PI3K/AKT signaling was primarily dependent on TLR4. In addition, administration of white ginseng oligopeptides to BALB/c mice enhanced the innate immune response, demonstrated by enhancement of the phagocytic capacity of macrophages and NK cell activity^37^.

White ginseng extracts promoted the maturation of DCs and upregulated the production of the proinflammatory cytokines TNF-α and IL-12 in human-derived peripheral blood mononuclear cells (PBMCs)^38^. Ginsan is an acidic polysaccharide isolated from white ginseng that has been studied for its immunomodulatory effects^39–41^. Cytokine-mediated major histocompatibility complex (MHC) class II expression and the costimulatory molecule CD86 are essential for upregulating T cell activation and maturation of DCs^42^. Ginsan was shown to induce the expression of CD86 and MHC class II markers in bone marrow-derived DCs (BMDCs) from mice and DCs derived from human monocytes in vitro^39,42^. Kim et al.^39^ demonstrated that ginsan induced the expression of the proinflammatory cytokines IL-12 and TNF-α and stimulated the proliferation of BMDCs harvested from C57BL/6 mice. Furthermore, ginsan-treated DCs induced the proliferation of allogeneic CD4+ T cells that markedly increased the production of both IFN-γ and IL-4^39^. These results suggest that ginsan may activate the costimulatory signal in DC-T lymphocyte interactions.

White ginseng extracts have also been reported to regulate NK cell activity. In vivo mouse studies revealed that treatment with white ginseng extracts induced the proliferation and cytotoxic activity of NK cells. With the enhanced activity of NK cells, the production of NK cell-secreted cytokines and IFN-γ expression were also increased^31,43^. The oral administration of white ginseng extracts enhanced the cytotoxic activity of NK cells isolated from wild-type B6 mice and BALB/c mice but not from IFN-γ knockout B6 mice, suggesting the involvement of IFN-γ in white ginseng’s immunostimulatory effects^44^. A randomized, double-blind clinical study on twenty healthy volunteers conducted by Scaglione et al.^45^ revealed that 8 weeks of ginseng extract consumption significantly increased NK cell activity compared to that of placebo-treated individuals. This study implies that the immunostimulatory effects of ginseng extracts may also be reproduced in humans. Treatment with the ginsenoside Rg1 stimulated the cytolytic activity of NK cells isolated from mice and augmented IL-1 production by macrophages^46^. Another mouse study also confirmed that Rg1 treatment enhanced the cytolytic activity of NK cells and restored the impairment of the immune response by cyclophosphamide treatment, suggesting that certain ginsenosides are at least partially responsible for the NK cell-activating effect of white ginseng^46,47^.

The immunomodulatory activity of white ginseng appears to also impact the adaptive immune system. In a mouse study, the oral administration of white ginseng extracts significantly increased the level of IgA in the spleen and serum^48^. Similarly, the administration of white ginseng extracts to mice for three consecutive days upregulated IgM and IgG production compared to that of the control group^49^. In addition, studies have shown the stimulatory effects of ginseng components on the production of antibodies against bacterial antigens. The effect of the ginsenoside Rg1 on antibody production was evaluated using *Toxoplasma gondii (T. gondii*) recombinant surface antigen 1 (rSAG1)^50^. Subcutaneous injection of Rg1 upregulated splenocyte proliferation and significantly enhanced the secretion of *T. gondii*-specific IgG antibodies^50^. When ginsan was administered to BALB/c mice immunized with *Salmonella*, the ginsan-treated mice secreted significantly greater amounts of serum IgG1, IgG2, and IgA against *Salmonella* than the control mice^51^. Studies have also demonstrated the immunostimulatory effects of white ginseng against viral infections. Su et al.^52^ studied the effects of the ginsenoside Re on the immune response against rabies virus (RV)-immunized BALB/c mice^52^. Treatment with Re significantly induced serum rabies-specific antibody production and enhanced CD4+ and CD8+ T cell expression. Compared to control BALB/c mice, readministered mice also displayed increased levels of the proinflammatory cytokines IL-4, IL-10, IL-12, and IFN-γ^52^.

Research has shown the involvement of white ginseng in controlling T cell activity. White ginseng extracts, polysaccharides, and ginsenosides were found to regulate T_H_ 1 and T_H_ 2 immune responses. White ginseng extracts induced a T_H_ 1-specific immune response, as demonstrated by enhanced proinflammatory IFN-γ and IL-12 cytokine production in human PBMCs^38^. Similarly, Lim et al.^31^ reported that white ginseng extract induced the expression of T_H_ 1 cytokines, TNF-α, IL-1α, and IL-23 and increased the phosphorylation of JNK1 and JNK2 in murine macrophage cells.

The T_H_ 1 axis-stimulating effect of white ginseng was further supported through a mouse model study. The administration of ginsan to BALB/c mice enhanced the proliferation and activity of T lymphocytes^53^. Treatment with ginsan also induced the production of IL-1, IL-6, IL-12, and IFN-γ^53^. Ginsenoside Rg1-treated BALB/c mice had enhanced T_H_ 2 immune activity, as demonstrated by increased CD4 + T lymphocyte counts and differentiation into T_H_ 2 cells^54^. In this study, Rg1 induced the mRNA expression of the T_H_ 2-specific IL-4 cytokine in CD4+ T cells while reducing the secretion of the T_H_ 1 cytokine IFN-γ^54^. Lee et al.^54^ reported that Rg1 stimulated the activity of CD4+ T cells and promoted differentiation into T_H_ 2 cells more than T_H_ 1 cells. In contrast to the promotion of T_H_ 1-specific immune responses in PBMCs by white ginseng extract^38^, the treatment of PBMCs with ginsenoside Rc and Rd compounds increased the differentiation and proliferation of T_H_ 2 cells more than those of T_H_ 1 cells^55^. The discrepancies between the effect of white ginseng extracts and single ginsenosides in mediating either a T_H_ 1 or T_H_ 2 immune response demonstrate that there is a complex interplay of constituents in white ginseng extracts and suggest that a more thorough study on individual components may be needed.

The contribution of white ginseng to controlling helper T cell responses has been translated into a protective effect against bacterial and viral infections. *Pseudomonas aeruginosa*-infected mice and live *Candida albicans (C. albicans)*-infected mice were treated with white ginseng extracts and ginsenoside Rg1, respectively^43,56^. Treatment with white ginseng extracts and Rg1 induced T_H_ 1 cell proliferation and the production of T_H_ 1-specific proinflammatory cytokines, including IFN-γ, IL-2, and TNF-α^43,56^. Pretreatment with Rg1 enhanced the protection of mice against *C. albicans*, as determined by the reduced number of colony-forming units (CFU) and prolonged survival of mice compared to those of the control mice^56^. Lee et al.^56^ also examined the relationship between increased T_H_ 1-specific IFN-γ production and enhanced protection against *C. albicans*. Anti-mouse IFN-γ antibody administered to Rg1-treated mice abrogated the protective effect of Rg1 against *C. albicans*, which reveals that the immunostimulatory effects of Rg1 are dependent on IFN-γ^56^. Additionally, the potential adjuvant role of white ginseng against porcine parvovirus (PPV), a virus causing reproductive failure in swine, was also studied^57^. The coadministration of the ginsenoside Rb1 with the PPV vaccine significantly stimulated IL-4 and IL-10 proinflammatory cytokine secretion in vaccinated mice^57^. White ginseng stem-leaf saponin extract, in combination with selenium (Se), was reported to significantly improve immune responses upon vaccination against pseudorabies virus (aPrV), a contagious herpesvirus in swine^58^. In this study, Wang et al.^58^ revealed that the adjuvant effect of this extract and immune response enhancement were dependent on the JAK-STAT pathway. Cotreatment with the extract and Se upregulated the production of both T_H_ 1-specific IgG2a and the cytokines IL-2, TNF-α, and IFN-γ as well as the T_H_ 2 response cytokines IL-4, IL-5, IL-6, and IL-10 and IgG1^58–60^. This study showed that the administration of white ginseng saponin extract may enhance both T_H_ 1 and T_H_ 2 immune responses^59,61^. The immunostimulatory and adjuvant effects of white ginseng demonstrated in these studies support the potential applications of white ginseng as a functional food.

### Red ginseng

The immunomodulatory effect of red ginseng has been primarily studied with respect to the innate immune system. In mice fed red ginseng, the size of the spleen and thymus and the number of white blood cells, including macrophages and NK cells, were increased^62^. In addition, red ginseng extract treatment of H1N1 virus-infected mice increased the expression of NKp46 on NK cells and upregulated IFN-γ production^63^. As a result of boosted NK cell activity, red ginseng was able to increase the survival rate of virus-infected mice. Research has shown that red ginseng can also activate macrophages. One study described that acidic polysaccharides from red ginseng increased NO production and the mRNA levels of iNOS in RAW264.7 macrophages through the regulation of extracellular signal-regulated kinase (ERK) and the JNK, AP-1, and NF-κB pathways^64^. Red ginseng acidic polysaccharides were also reported to induce the production of cytokines such as IL-1 and IL-6 in macrophages^64^. However, treatment with a combination of red ginseng acidic polysaccharide and IFN-γ significantly increased the production of IL-1 and IL-6 as well as TNF-α through the activation of NF-κB^64^. Therefore, the consumption of red ginseng may enhance innate immunity by controlling the activity of NK cells and macrophages.

Red ginseng extracts have also been reported to inhibit bacterial and viral infections by stimulating the adaptive immune response. The effects of red ginseng on the infection of influenza viruses, including influenza virus A/PR8, H1N1 virus, and H9N2 virus, were investigated^63,65–67^. The administration of red ginseng extract to mice infected with influenza A/PR8 significantly increased the production of serum IgA and all IgG subtypes^65^. Treatment of human PBMCs with red ginseng extracts upregulated the expression of CD25 and CD69, which are responsible for the proliferation of CD3+ T cells^63^. In the same study, the administration of red ginseng extracts to H1N1 virus-infected mice ameliorated H1N1 virus-induced lytic gene expression and viral plaque accumulation and increased the survival rate of mice^63^. Yoo et al.^68^ reported that oral administration of red ginseng extracts had antiviral effects against H1N1 and H3N2 influenza virus in mice^68^. Oral administration of red ginseng extracts was found to enhance cross-protection against antigenically distinct H1N1 and H3N2 influenza viruses. Treatment of H1N1 virus-infected mice with red ginseng extract resulted in a significant reduction in lung viral titers and increased expression of the antiviral cytokine IFN-γ compared to those of the untreated mice^68^.

The potential adjuvant properties of red ginseng ginsenosides on cellular and humoral immune responses in ovalbumin (OVA)-immunized mouse models have been studied^69–72^. The red ginseng ginsenosides Rg1, Rd, and Re induced T_H_ 1 and T_H_ 2 lymphocyte proliferation and enhanced OVA-specific IgG antibody production against OVA in ICR and BALB/c mice^69–72^. Rg1, Rd, and Re significantly induced the expression of IL-4, IL-10, IFN-γ, IL-5, and IL-2, as well as the production of IgG1 and IgG2a^69–72^. Su et al.^71^ studied the molecular mechanisms involved in the adjuvant effects of Rg1 and Re using OVA-immunized C3H/HeB mice and TLR-4 defective C3H/HeJ mice. Both Rg1 and Re stimulated the activation of NF-κB and the expression of proinflammatory cytokines^73^ in C3H/HeB mice but not in TLR-4 knockout C3H/HeJ mice^71^. This result suggests that T_H_ 1/T_H_ 2 immune enhancement and adjuvant effects of red ginseng ginsenosides are dependent on the TLR-4 signaling pathway^71^. The immunostimulatory and adjuvant properties of red ginseng against viral and bacterial infections demonstrate the potential applications of red ginseng as a functional food to improve the host immune response. In a randomized, double-blind, placebo-controlled clinical trial on 100 healthy subjects, the red ginseng intake group had significantly increased T cell and B cell counts compared to those of the placebo group at week 8^74^. Secondary efficacy evaluation measured by vital signs and hematological tests confirmed that the administration of red ginseng extracts did not cause any adverse responses^74^. A randomized, double-blind, placebo-controlled clinical trial was performed on 100 healthy subjects for 12 weeks to investigate the effects of red ginseng extract on acute respiratory illness (ARI), a self-limiting viral disease caused by viruses including rhinovirus and coronavirus^75^. This study reported that the red ginseng-treated group had a lower ARI frequency rate, lower symptom score, and shorter symptom duration than the placebo group over the 12-week study^75^. These clinical studies support the hypothesis that the consumption of red ginseng may improve immunity in healthy human subjects and promotes the potential applications of red ginseng extracts for use as a complementary treatment of influenza A virus^74,75^.

## Mushrooms

The mushroom is a fruiting body of a fungus, which is produced by more than 1000 different species. Mushrooms have been widely consumed worldwide as a food ingredient, and various bioactivities of mushrooms have been reported^76^ (Table 2).

**Table 2:** The immunostimulatory functions and mechanism of mushrooms and *B. subtilis chungkookjang*.

Source	Cell type	Function	Model	Reference
*A. blazei*	Splenocytes	IFN-γ, IFN-γ-IP-10↑ IL-1β, IL-4, IL-6↑ Cell proliferation↑	Animal	^77,79^
	PBMCs	Phagocytosis↑	Animal	^79^
	NK cells	IFN-γ↑	Animal	^78,79,136^
*G. lucidum*	Splenocytes	IFN- γ, IL-2, Cell proliferation↑	Animal	^82,83^
	Macrophages	TNF-α, IL-6, IL-1β↑↑ Phagocytosis, NO↑	In vitro, animal	^81,83^
	NK cells	Cytotoxicity↑ IL-2, IFN- γ, CD56+ cells↑	Animal, human	^83,84^
*G. frondosa*	Splenocytes	IFN- γ, TNF-α, IL-12 p70, IL-18↑	Animal	^86,87^
	Macrophages	IL-12↑	Animal	^87^
	NK cells	TNF-α, Cytotoxicity↑	Animal	^87,91^
	DCs	IL-12 p70↑	Animal	^90^
	T cells	TNF-α, IFN-γ↑ Foxp3+ CD4+ cells↓	Animal, human	^85,90,92^
	APCs	IFN-γ, IL-12↑	Animal	^89,91^
*B. subtilis chungkookjang* (γ-PGA)	PBMCs	Cytotoxicity↑	Human	^103^
	Macrophages	TNF-α, IFN-γ-IP-10↑ NLRP3, IL-1, IL-6, IL-10↑	Ex vivo	^98,101^
	NK cells	Cytotoxicity, IFN-γ↑	Animal	^99,104^
	DCs	IL-12↑	Ex vivo	^100^
	Natural killer dendritic cells	IL-12, IL-23, IFN-γ↑ Perforin, FasL, TRAIL↑	Ex vivo, animal	^100^
	T cells	IFN-γ-expressing CD8+ cells↑	Animal	^104^

### *Agaricus blazei* (*A. blazei*)

*A. blazei* is known for its immunostimulatory properties, which include the enhancement of cytokine production and induction of immune cell proliferation. A study showed that the stimulation of immune responses may have generated antitumor effects in mice. Spleen cells from *A. blazei*-treated tumor-bearing mice showed increased IFN-γ production and IFN-γ-inducible protein (IP-10) mRNA expression compared to those of spleen cells from the control mice. In addition, mice treated with *A. blazei* extracts showed decreased tumor size and weight and increased expression of CD69, which is an activation marker of infiltrating T cells in tumors^77^. These results could imply that *A. blazei* extracts may activate immune cells to promote tumor rejection. In addition, administration of *A. blazei* extracts increased NK cell activity in a Meth A-bearing mouse model^78^ in a dose-dependent manner and promoted the phagocytic activity of macrophages in a murine leukemia BALB/c mouse model^79^. These changes in immune cell activity were accompanied by a decrease in tumor size and weight^78^. In addition to its effect on tumors, the potential therapeutic implication of *A. blazei* against cerebral malaria (CM) was examined^80^. CM is caused by *Plasmodium berghei*, which induces lipid peroxidation through the release of ROS. The administration of *A. blazei* for three days significantly reduced ROS activity, inhibited lipid peroxidation, and ameliorated parasite infection severity in mice^80^. *A. blazei*-treated mice also had reduced cytokine production compared to that of untreated mice. Mice treated with *A. blazei* exhibited increased parasitemia levels, elevated survival rates and reduced weight loss, which led to preventive effects on CM development^80^.

### *Ganoderma lucidum* (*G. lucidum*, reishi)

*G. lucidum* has shown immunostimulatory effects that might be translated into cancer therapeutic activity. A study examining the effect of *G. lucidum* on macrophages in vitro confirmed that TNF-α and IL-6 secretion was significantly stimulated in a dose-dependent manner compared with that in untreated control macrophages^81^. In this study, the researchers identified that the effect of *G. lucidum* on macrophages occurred through the upregulation of MAPK signaling pathways, including ERK1/2, p38, and JNK, in murine resident peritoneal macrophages. When tumor-bearing mice were treated with *G. lucidum*, there was an increase in the concentration of IFN-γ and IL-2 in the blood as well as in the cytotoxicity of NK cells^82^. This study showed upregulation of NF-κB expression in the spleen of mice treated with *G. lucidum*, suggesting that the immunomodulatory function of *G. lucidum* occurs be through the activation of the NF-κB signaling pathway^82^. In addition, proliferation of spleen lymphocytes was promoted, and NK cell activity and macrophage phagocytic activity were augmented^83^. In a clinical study, patients with advanced lung, colon, or breast cancers treated with *G. lucidum* for 12 weeks showed increased levels of IL‐2, IL‐6, and IFN‐γ in their plasma and an increased number of CD56+ cells. Additionally, *G. lucidum* treatment induced a significant increase in the mean NK cell activity compared with the baseline (34.5% ± 11.8% vs. 26.6% ± 8.3%) in advanced‐stage cancer patients^84^. In summary, *G. lucidum* treatment induces changes in cytokine production and immune cell activity, which could contribute to suppressing tumor growth in vivo.

### *Grifola frondosa* (maitake D)

A polysaccharide designated the maitake D fraction, extracted from *Grifola frondosa* has been reported to exert antitumor effects by activating macrophages and T cells and increasing the expression of T_H_ 1 cytokines while simultaneously suppressing the production of the T_H_ 2 cytokine IL-4^85^. As T_H_ 2 activation decreases T_H_ 1 levels, by suppressing T_H_ 2 responses, *G. frondosa* establishes a T_H_ 1-dominant phenotype^86^. When *G. frondosa* was administered to mice, the expression of IFN-γ and IL-12 in antigen-presenting cells (APCs) was increased, and the activities of NK cells and macrophages were augmented^87–89^. Orally administered *G. frondosa* fraction inhibited tumor growth in various models while stimulating immune responses. It has been revealed that the *G. frondosa* fraction increases the number of IFN-γ-expressing CD4+ and CD8+ T cells^88^ and IL-12 p70 production through the regulation of dectin-1 in DCs in colon cancer models^90^. Intraperitoneal administration of the maitake D fraction 2 days before tumor implantation significantly inhibited lung metastasis. Moreover, the *G. frondosa* fraction increased the production of IL-12 in APCs and the cytotoxicity of NK cells^91^. The maitake D fraction directly stimulated DC maturation through a C-type lectin receptor dectin-1 pathway. Oral administration of the maitake D fraction was reported to increase systemic tumor antigen-specific T cell responses and T cell infiltration into tumor sites. Oral intake of the maitake D fraction also reduced the number of regulatory T cells and myeloid-derived suppressor cells^90^. In a clinical study (a phase II study examining the effects of *G. frondosa* on myelodysplastic syndromes, MDSs), *G. frondosa* β-glucan consumption improved neutrophil and monocyte function in lower-risk MDS patients. Moreover, treatment with *G. frondosa* extract increased the ROS response to *Escherichia coli (E. coli)* ex vivo, indicating that *G. frondosa* might enhance the immune response to bacterial infections in MDS patients. Similarly, in a *G. frondosa* phase I/II trial of breast cancer patients, the intermediate dose (5–7 mg/kg/day) was associated with increased TNF-α and IFN-γ production by T cells^92^.

Mushrooms are known to exert immunomodulatory and antitumor effects through the activation of immune cells. Studies have been conducted to examine cytokine production, cell activation, and effects on tumors upon treatment with *A. blazei*, *G. lucidum*, and *G. frondosa*. In most studies of these mushrooms, the production of IFN-γ, along with that of IL-1, IL-6, IL-8, IL-12, and TNF-α, was increased after treatment. The polysaccharide fraction, mainly β-glucans, is the component that is known to be responsible for the immunomodulatory effects exerted by mushrooms, and its effect appears to depend on the structural characteristics of β-glucans^93^. The receptors of these β-glucans include dectin-1, TLR, and CR3 (also known as CD11b/CD18)^94–96^. Dectin-1 and TLR, which are expressed on macrophages, bind to β-glucans, which are pathogen-associated molecular patterns, thereby inducing macrophage activation and promoting the production of inflammatory cytokines. In addition, β-glucans induce the functional maturation of DCs and the production of IL-12 and IFN-γ, indirectly promoting the activation of T cells. β-glucans also bind to CR3, which is highly expressed on neutrophils, monocytes, and NK cells, and prime these cells to bind to inactivated complement 3b (iC3b), which directs β-glucan-activated cells to induce the lysis of iC3b-coated cells^94,96^. Most studies investigating the immunomodulatory effects of mushrooms have been conducted using mushroom extracts. Therefore, studies using highly purified β-glucans could benefit from understanding a more precise mechanism by which β-glucan and mushrooms affect immunity.

## *Bacillus subtilis* chungkookjang (poly-γ-glutamate, γ-PGA)

*Bacillus subtilis* subsp. *chungkookjang* is found in chungkookjang, a traditional Korean fermented soybean food. *Bacillus subtilis* subsp. *chungkookjang* naturally produces an edible biomaterial called poly-γ-glutamate (γ-PGA), which is a polymer with a γ-amide bond between D- or L-glutamate with a molecular weight of 1000 kDa or more^97^. The immunomodulatory functions of γ-PGA and its potential use as a drug or dietary supplement have been studied in cancer and virus infection models (Table 2). γ-PGA induced the expression of proinflammatory cytokines, including IL-1β, TNF-α, IL-6, and NLRP3, through its interaction with TLR4 in BMDMs^98^. The treatment of γ-PGA stimulated the production of IFN-γ in NK cells and enhanced NK cell cytotoxicity in a melanoma mouse model^99^. Treatment with high-molecular-mass (2000 kDa) γ-PGA led to an increase in the number of NK cells, with a concomitant reduction in tumor size, in a lung cancer model^99^. Additionally, TNF-α, IFN-γ, and IL-12 secretion from activated NK DCs were induced by γ-PGA^100^. Moreover, a study demonstrated the crucial involvement of TLR4 in γ-PGA-mediated antitumor immunity using MyD88 knockout and TLR4-defective mice^101^. In this study, Lee et al. reported that the stimulatory potency toward macrophages and immature DCs as well as the antitumor effect of γ-PGA was lost when TLR4 signaling was genetically blocked, revealing an important functional target of γ-PGA. In a multicenter, randomized, double-blind clinical trial of 195 patients with cervical intraepithelial neoplasia (CIN) 1, 42.4% of the patients who received oral administration of γ-PGA showed histological remission of CIN 1, compared to 27.1% of the control subjects^102^. High-risk human papillomavirus (HPV) clearance was observed in 43.5% of the patients receiving γ-PGA, whereas 26.7% clearance was found in the control subjects^102^. However, while there was a mild increase in NK cell activity induced by γ-PGA administration at week 8, the activity was not higher at week 12^102^. On the other hand, in a randomized double-blind placebo-controlled clinical study with healthy volunteers, oral administration of γ-PGA for 8 weeks caused higher NK cell cytotoxicity compared to that of the placebo group when examined at the end of the study^103^. The discrepancy in NK cell activity between the two human studies implies that the effect of γ-PGA may not be sustained for a long time after cessation of consumption because the γ-PGA administration period was 4 weeks shorter in the CIN 1 patient study than in the study conducted with healthy volunteers. In another study focusing on the anti-infection effect, intranasal γ-PGA administration protected against H1N1 influenza A virus in vivo by inhibiting viral infection, which in turn led to an increase in the survival rate of infected mice^104^. Moreover, influenza-specific cytotoxic T cell activity increased upon γ-PGA treatment^101,104^.

## Chlorella

Chlorella is a unicellular green algae widely used as a functional food and nutraceutical due to its rich content in proteins, dietary fibers, vitamins, minerals, and other bioactive compounds^105^. *Chlorella vulgaris* and *Chlorella pyrenoidosa* are the most studied species among the chlorella for use as dietary supplements^106^.

### *Chlorella vulgaris* (*C. vulgaris*)

*C. vulgaris* has been reported to enhance immunity in animal models as well as human studies and may improve defense against microbial infections (Table 3). In a cyclophosphamide-mediated immunosuppression mouse experiment, dried *C. vulgaris* rescued the cyclophosphamide-induced downregulation of IL-2, TNF-α, IFN-γ, and IL-12 expression. More importantly, *C. vulgaris* treatment was able to increase the cytotoxic activity of NK cells in cyclophosphamide-treated mice. In addition, the proliferation of lymphocytes and phagocytic activity of macrophages was also increased by *C. vulgaris*^107^. In vitro examination revealed an increase in IFN-γ and IL-2 levels in MOLT-4 cells^108^. In an 8-week randomized, double-blinded, placebo-controlled study, treatment of healthy participants with dried *C. vulgaris* extract tablets resulted in higher NK cell activity than that in the placebo-treated subjects. Additionally, serum cytokine concentrations, especially those of IFN-γ and IL-1β, were significantly increased in the participants who received chlorella supplementation, suggesting a potential immunostimulatory effect in healthy individuals^109^. When chlorella was fed to chickens as a supplement, the concentrations of plasma IgG and IgM increased compared to those in the group without supplementation^110^. Interestingly, the number of lymphocytes and white blood cells increased in broiler chickens treated with 1% fresh liquid chlorella but not in those treated with 1% dried chlorella powder^111^. This finding implies that while chlorella might provide immunomodulatory effects in vivo, the type of supplement may be important in determining bioactivity.

**Table 3:** The immunostimulatory functions and mechanism of chlorella.

Source	Cell type	Function	Model	Reference
*C. vulgaris*	White blood cells	Cell number↑	Animal	^111^
	Peritoneal adherent cells	IL-1α, IL-12, GM-CSF, MIP, TNF- α↑	Animal	^113^
	Splenocytes	Cell proliferation↑ IL-2, IL-12, TNF-α, IFN-γ↑	Animal	^107,113^
		IL-12 p40↑	Animal	^115^
	Macrophages	Phagocytosis↑ Cell proliferation↑	Animal	^107^
		HLA-DA, HLA-DC↑ TNF-α, IL-1β, CD80, CD86↑	Animal, human	^117^
	NK cells	Cytotoxicity↑	Animal, human	^107,109^
	T cells	Cell proliferation↑ IFN-γ, IL-2, IL-4↑	Animal	^108^
	Blood	IFN-γ, IL-1β, IL-2↑ IgA, IgG, IgM↑	Animal, human	^108–111^
*C. pyrenoidosa*	Blood	Antibody production↑	Human	^119^
	Macrophages	IL-1β, TNF- α↑	In vitro	^118^
	Macrophages	Phagocytosis, NO↑	In vitro	^116^
	Breast milk	IgA ↑	Human	^120^

*C. vulgaris* has been reported to enhance antibacterial immunity against several types of bacterial infections. *C. vulgaris* water extract lowered the number of bacteria in the peritoneal cavity or spleen of mice after *Listeria monocytogenes (L. monocytogenes)* infection, which appeared to occur through augmentation of the T cell-mediated immune response^112^. Further studies demonstrated that the resistance to *L. monocytogenes* conveyed by *C. vulgaris* involved the activation of the T_H_ 1 response driven by IFN-γ and IL-12^113,114^. *C. vulgaris* also augmented resistance against intraperitoneal *E. coli* infection in rats. Oral administration of *C. vulgaris* decreased the viable number of bacteria in the blood, spleen, and liver while increasing the activity of polymorphonuclear leukocytes^112^. Treatment with *C. vulgaris* extracts also improved antitumor activity in mice by inducing the production of IL-12 p40. To investigate which biological pathway mediates the antitumor activity of C. *vulgaris*, spleen-adherent cells derived from TLR4-lacking C3H/HeJ mice and TLR2 knockout mice were separately treated with *C. vulgaris* extract. IL-12 p40 cytokine production was significantly impaired in C. *vulgaris*-stimulated spleen-adherent cells isolated from TLR2 knockout mice compared to that in corresponding cells from WT mice. This study suggests that the immunomodulatory effects of *C. vulgaris* are dependent on TLR2 signaling^115^. These recent findings reveal the potential of *C. vulgaris* as a potent immunostimulatory agent. Further research on the biological pathways involved in the immunomodulatory effects of *C. vulgaris* would provide a better understanding and encourage the potential uses of *C. vulgaris* to improve the immune system.

### *Chlorella pyrenoidosa* (*C. pyrenoidosa*)

Immunomodulatory activities of *C. pyrenoidosa* have been found mainly in macrophages and humoral immune responses (Table 3). The effect on macrophage activation was measured based on phagocytic activity and intracellular NO generation in murine macrophage cell lines. The phagocytic activity of macrophages was elevated by hot water extracts of *C. pyrenoidosa*. These extracts also enhanced macrophage proliferation and NO generation^116^. Treatment with hot-water-soluble polysaccharides of *C. pyrenoidosa* induced the expression of human leukocyte antigen (HLA)-DA and HLA-DC in human blood monocyte-derived macrophages^117^. The expression of the proinflammatory cytokines TNF-α and IL-1β and the production of the costimulatory molecules CD80 and CD86 were found to be upregulated by *C. pyrenoidosa*^117^. In this study, Hsu et al.^117^ suggested that the immunostimulatory effects of *C. pyrenoidosa* were mediated by the TLR4-mediated signaling pathway. In a similar study, the polysaccharide fraction of *C. pyrenoidosa* was also found to increase the expression of TNF-α and IL-1β mRNA and cause NF-κB activation in THP-1 cells^118^. These findings encourage further research on the potential use of *C. pyrenoidosa* as an agent to improve immune responses.

The immune-enhancing function of *C. pyrenoidosa* supplements was examined in a clinical experiment on healthy subjects above 50 years of age who have treated with an influenza A vaccine. No meaningful results were obtained, but subjects aged 50–55 years exhibited increased anti-influenza A antibody production^119^. When *C. pyrenoidosa* tablets were administered to pregnant women, concentrations of IgA significantly increased in breast milk compared to those in the control group. This finding implies that *C. pyrenoidosa* supplementation during pregnancy could reduce the probability of infection in nursing infants by increasing IgA levels in breast milk^120^. In addition, oral administration of *C. pyrenoidosa* powder to rats enhanced the production of IgM in spleen and mesenteric lymph node lymphocytes and increased serum concentrations of IgM and IgG^121^.

## *Lactobacillus plantarum* (*L. plantarum*)

Probiotics are widely used as health-promoting food ingredients due to their various bioactivities^122^. Recent studies have shown that not only live probiotics but also heat-killed probiotics or fractionated cellular components (exopolysaccharides, EPSs) of the bacteria can have beneficial effects on the host immune system^123^. Among various probiotics, *L. plantarum* was chosen because it is one of the most-researched species due to its immunomodulatory functionality (Table 4).

**Table 4:** The immunostimulatory functions and mechanism of *L. plantarum*.

Source*	Cell type	Function	Model	Reference
*L. plantarum* AYA	DCs	IL-6, TGF-β, IgA ↑	Animal	^132^
*L. plantarum* nF1	Blood	IgA, IgG↑	Animal	^24^
	Splenocytes	IL-4, IL-5, IL-17A, IL-12, TNF- α ↑		
	Macrophages	NO, IL-6, TNF- α ↑		
*L. plantarum* YU	Blood	IgE ↑	Animal	^130^

It has been reported that heat-killed and micronized *L. plantarum* and EPS from *L. plantarum* enhance phagocytic activity and cytokine production (e.g., TNF-α and IL-6) in macrophages through the activation of the NF-κB and MAPK signaling pathways^124–127^. A study reported that micronized and heat-killed *L. plantarum* LM1004 resulted in a marked increase in TLR2 mRNA expression levels in macrophages. Moreover, various types of *L. plantarum*, such as heat-killed *L. plantarum* Ln1, live *L. plantarum* nF1 and dead nanosized *L. plantarum* nF1, have been reported to enhance NO production and iNOS expression levels in macrophages^24,124,128^. In addition, mouse DCs treated with *L. plantarum* showed upregulated levels of cytokines, including IL-10, IL-12 p40, IL-20 p70, and TNF-α, through the upregulation of the NF-κB and MAPK signaling pathways^129^.

*L. plantarum* is known to modulate the adaptive immune system through the regulation of T cells as well as IgA production. Dead *L. plantarum* has been reported to promote T_H_ 1 (TNF-α and IL-12 p70) and T_H_ 17 (IL-6 and IL-17A) responses rather than T_H_ 2 (IL-4 and IL-5) responses in splenocytes isolated from normal male C57BL/6J mice^24^. In an OVA/alum-immunized mouse model, *L. plantarum* YU-treated mice produced higher levels of IFN-γ, while the levels of the T_H_ 2-related cytokines IL-4 and IL-10 were not significantly different than those in the control mice^130^. In addition to T cell regulation, *L. plantarum* can enhance IgA production. IgA production was significantly stimulated by oral administration of *L. plantarum* YU^130^. Additionally, in a cyclophosphamide-induced immunosuppression mouse model, the number of cells secreting IgA and the level of secretory IgA (sIgA) in the small intestine was increased by the administration of *L. plantarum*^131^. Treatment with *L. plantarum* in mice challenged with intranasal inoculation of influenza A virus (IFV) enhanced IFV-specific sIgA levels in bronchoalveolar lavage fluid (BALF). As a result, viral proliferation and the viral titer in the lung were strongly suppressed by the administration of *L. plantarum*^130,132^.

The immunomodulatory effects of *L. plantarum* were also demonstrated in a clinical study. The study showed that *L. plantarum* restored the number of regulatory T cells in the serum that were suppressed by nonsteroidal anti-inflammatory drugs (NSAIDs). *L. plantarum* also enhanced memory responses of T cells against tetanus toxoid (TT)-antigen and upregulated expression of genes associated with T and B cell function in the small intestinal mucosa^133^. *L. plantarum* was also found to improve immune responses in older subjects. In this study, researchers demonstrated that a high dose of *L. plantarum* increased the composition of activated NK cells, whereas a low dose of *L. plantarum* increased activated T cells, B cells, and APCs^134^. This clinical study revealed that the concentration of *L. plantarum* may elicit different immune responses, suggesting that the concentration may be an important factor when consuming *L. plantarum* to control immune function. These various previous studies have shown that various types of *L. plantarum* can activate both the innate immune response and the adaptive immune response.

## Conclusion

In this review, we summarized food materials with immunomodulatory effects that have been studied in vitro, in vivo, and in clinical models (Tables 1–4). The immune system is essential for providing protection against pathogens. Hosts with compromised or weakened immune function are more vulnerable to cancer or infectious diseases. The consumption of functional foods such as ginseng, mushroom, poly-γ-glutamate, chlorella, and *L. plantarum* may improve the immunological defense system, which in turn could help protect the body from diseases. Further identification of novel functional foods with immunomodulatory activity and study of their molecular mechanisms could aid in improving public health.
